# MRI Evidence for Altered Venous Drainage and Intracranial Compliance in Mild Traumatic Brain Injury

**DOI:** 10.1371/journal.pone.0055447

**Published:** 2013-02-06

**Authors:** Andreas Pomschar, Inga Koerte, Sang Lee, Ruediger P. Laubender, Andreas Straube, Florian Heinen, Birgit Ertl-Wagner, Noam Alperin

**Affiliations:** 1 Institute of Clinical Radiology, University of Munich – Grosshadern Campus, Ludwig-Maximilians-University Munich, Munich, Germany; 2 Department of Radiology, Miller School of Medicine, University Miami, Miami, Florida, United States of America; 3 Institute of Medical Informatics, Biometry, and Epidemiology (IBE), Ludwig-Maximilians- University Munich, Munich, Germany; 4 Department of Neurology, Ludwig-Maximilians-University Munich, Munich, Germany; 5 Department of Pediatric Neurology and Developmental Medicine, Dr. von Hauner’s Children’s Hospital, Ludwig-Maximilians-University Munich, Munich, Germany; University of Maryland, United States of America

## Abstract

**Purpose:**

To compare venous drainage patterns and associated intracranial hydrodynamics between subjects who experienced mild traumatic brain injury (mTBI) and age- and gender-matched controls.

**Methods:**

Thirty adult subjects (15 with mTBI and 15 age- and gender-matched controls) were investigated using a 3T MR scanner. Time since trauma was 0.5 to 29 years (mean 11.4 years). A 2D-time-of-flight MR-venography of the upper neck was performed to visualize the cervical venous vasculature. Cerebral venous drainage through primary and secondary channels, and intracranial compliance index and pressure were derived using cine-phase contrast imaging of the cerebral arterial inflow, venous outflow, and the craniospinal CSF flow. The intracranial compliance index is the defined as the ratio of maximal intracranial volume and pressure changes during the cardiac cycle. MR estimated ICP was then obtained through the inverse relationship between compliance and ICP.

**Results:**

Compared to the controls, subjects with mTBI demonstrated a significantly smaller percentage of venous outflow through internal jugular veins (60.9±21% vs. controls: 76.8±10%; p = 0.01) compensated by an increased drainage through secondary veins (12.3±10.9% vs. 5.5±3.3%; p<0.03). Mean intracranial compliance index was significantly lower in the mTBI cohort (5.8±1.4 vs. controls 8.4±1.9; p<0.0007). Consequently, MR estimate of intracranial pressure was significantly higher in the mTBI cohort (12.5±2.9 mmHg vs. 8.8±2.0 mmHg; p<0.0007).

**Conclusions:**

mTBI is associated with increased venous drainage through secondary pathways. This reflects higher outflow impedance, which may explain the finding of reduced intracranial compliance. These results suggest that hemodynamic and hydrodynamic changes following mTBI persist even in the absence of clinical symptoms and abnormal findings in conventional MR imaging.

## Introduction

Traumatic brain injury (TBI) affects over 1.4 million individuals annually in the United States alone [1]. The majority of TBI are classified as mild traumatic brain injury (mTBI) defined as a blunt head trauma resulting in transient confusion, disorientation, impaired or loss of consciousness lasting 30 minutes or less in combination with a number of unspecific neurological and cognitive symptoms [1]–[3]. Despite the lack of cerebral abnormalities on conventional anatomical imaging such as CT or MRI, patients experience a great variety of long-term neurological symptoms such as orthostatic hypotension, headache, disturbed concentration and fatigue resulting in functional and financial problems that impact the healthcare system and society in general.

The pathophysiology associated with symptoms reported after mTBI still remains to be further elucidated. There is histological evidence for diffuse morphological changes after mTBI in humans including microscopic axonal injury leading to Wallerian degeneration, transaction, and microglial clusters [4], [5]. Advanced MR based imaging and spectroscopy techniques, such as functional MRI (fMRI) [6], [7], diffusion tensor imaging (DTI) [8]–[10], and MR spectroscopy [11] have shown to be more sensitive in detecting alterations in the brain following mTBI. For example, increased fractional anisotropy (FA) and decreased radial diffusivity in the white matter e.g. the corpus callosum were detected using DTI, suggestive of axonal cytotoxic edema [8]–[10]. Recent fMRI studies found a more disperse brain activation pattern with a pronounced activation in the dorsolateral prefrontal cortex and the hippocampus after mTBI [6], [7]. MRS demonstrated widespread cellular metabolic dysfunction including a decrease of N-acetyl aspartate and an increase in total choline. These changes correlated with neuropsychological parameters after mTBI [11].

Investigations of the potential impact of mTBI on the microvasculature in humans are scarce. Morphological changes in the cerebral microvasculature were documented in a nonhuman primate animal model of mTBI, where a persistent increase in endothelial projections in the arterioles and venules throughout the brain was found after lateral head acceleration [12]. Though, these morphological changes cannot be directly visualized with current imaging methods, they may lead to alterations in the biomechanical properties of the cerebral microvasculature, which in sum, could affect cerebral hemodynamics [13].

A link between cerebral vascular compliance and venous drainage is implied from differences in venous drainage patterns and venous outflow pulsatility between upright and supine postures [14]. In the supine position, the internal jugular veins (IJVs) are the dominant pathway of cerebrovenous drainage, while in the upright posture venous drainage is shifted to secondary pathways. These secondary venous pathways have been described as early as 1957 by Batson [15]. Although secondary venous drainage is highly variable [16], [17] studies have consistently identified three main non-jugular venous drainage pathways: (a) internal vertebral venous plexus i.e. the epidural veins (EV), (b) vertebral artery venous plexus i.e. the vertebral veins (VV), and (c) the more posterior deep cervical veins (DCV). Changes in venous drainage pathways are also associated with changes in intracranial compliance and pressure [14], [18]. Therefore, the aims of this study were (1) to evaluate craniocervical venous drainage and (2) to assess potential changes in intracranial compliance following mTBI. Venous drainage patterns are assessed qualitatively with MR venography and quantitatively with measurements of volumetric flow rates with MR velocity-encoded phase contrast imaging. MRI estimates of intracranial compliance and pressure (MR-ICP) are obtained using an experimental non-invasive technique that estimates compliance and pressure from measurements of arterial inflow, venous outflow and CSF flow to and from the cranial vault during the cardiac cycle [19].

## Subjects and Methods

### Ethics Statement

Approval from the Ethics Committee of the Medical Faculty at Ludwig-Maximilians-University, Munich, Germany was obtained prior to the study and all study participants provided written informed consent.

### Subjects

Thirty subjects were included in the study. The subjects were recruited among colleagues and co-workers in our university hospital and through an announcement in the hospital’s internal clinical bulletin. All subjects were questioned in detail regarding general and neurological health and completed a detailed standardized questionnaire based on the criteria for mTBI of the Centers for Disease Control (CDC). The questionnaire asked for history and description of head trauma, time since trauma, symptoms following the trauma such as headache, nausea, vomiting, dizziness, neck pain, retrograde or anterograde amnesia, fatigue, irritability to light and sound, duration of each symptom, problems concentrating or learning difficulties after the trauma.

Inclusion criteria were a mTBI even fulfilling the CDC criteria that occurred at least 6 months or longer prior to the study, full recovery from the clinical signs and symptoms associated with mTBI within one week of the trauma, absence of chronic and ongoing symptoms following the trauma, and an age above 18 years. Inclusion criteria for the control cohort were an age and gender match with a subject in the mTBI cohort and the absence of any history of traumatic brain injury or whiplash trauma. Exclusion criteria for the mTBI cohort included a history of mTBI less than 6 month prior to inclusion into the study. Exclusion criteria for both cohorts consisted of a history of neurological or psychiatric disorders, including learning disabilities or of other chronic medical conditions (including hypertension, cardiac conditions, diabetes mellitus or cancer) or medication intake (other than oral contraceptives in female subjects) and MR-related contraindications, including cardiac pacemakers, other metallic implants or claustrophobia. According to the guidelines of the Centers for Disease Control and Prevention [1], mTBI is assumed if head injury resulting from blunt trauma, acceleration or deceleration forces was reported and additionally one or more of the following conditions were associated with the head injury: any period of observed or self-reported transient confusion, disorientation, impaired consciousness, or loss of consciousness lasting 30 minutes or less, any period of observed or self-reported dysfunction of memory (amnesia) around the time of injury, observed signs of other neurological or neuropsychological dysfunction, such as nausea, vomiting, headache, dizziness, irritability, fatigue or poor concentration. Fifteen subjects (4 female; range: 20–49 years; mean 27.4±7.1 years) reported a mild traumatic brain injury either due to a car accident possibly combined with whiplash (3 subjects), or due to a fall related impact to the head (12 subjects). Ten subjects reported unconsciousness lasting less than 30 min. All 15 subjects reported symptoms directly following the mTBI (e.g. headache, nausea, vomiting, dizziness, neck pain, retrograde and anterograde amnesia) lasting no longer than 2 days. mTBI related symptoms of all studied subjects resolved within 2 days after the trauma and all subjects were asymptomatic at the time of our study. Time since trauma ranged from 6 months to 29 years (mean 11.4 years). The fifteen age- and gender-matched subjects with no history of trauma were scanned with the same protocol (4 female; range: 18–48 years; mean 27.0±7.2 years).

### Imaging Data Acquisition

Subjects were imaged in supine position using a 3 Tesla MR scanner (Verio, Siemens Healthcare, Erlangen, Germany) with a 12 channel phased array head and neck coil. The MRI study protocol included conventional anatomical brain sequences such as FLAIR- and 3D T1-weighted images to rule out any structural pathology. An axial 2D TOF MR venography was added to image the veins in the infratentorial and upper cervical region for assessment of the venous drainage pathways. Imaging parameters included FoV of 160 mm, slice thickness of 2 mm, matrix size of 256×244, TR of 23 ms, TE of 5.4 ms, and a flip angle of 45 deg.

Two retrospectively-gated velocity encoding (VENC) cine phase contrast scans, one with a high VENC of 70–90 cm/sec) and one with a low VENC of 7–9 cm/sec were added to measure blood and CSF flow rates to and from the cranium. Imaging planes for the blood and CSF flow measurements were placed at the height of the dens axis perpendicular to the internal carotid and vertebral arteries, and at the mid C2 level, respectively, as described by Tain et al. [20]. Imaging parameters of the phase contrast scans included a FoV of 140 mm, matrix size of 256×179, slice thickness of 4 to 6 mm, and flip angle of 20 deg. Minimum TE and TR were used for maximal temporal resolution. One average and two views per segment were used to keep acquisition time around 1.5 minutes per scan (approximately 90 cardiac cycles).

### Assessment of Venous Drainage Patterns

Venous drainage was assessed qualitatively and quantitatively. Qualitative visual assessment was obtained using 3D-maximum intensity projection (MIP) models of the MRVs. The MRV source images and the 3D reconstructed models were inspected to determine the degree of secondary venous outflow by a board certified neuroradiologist who was blinded to the subjects’ status using the following scale: 1 - no; 2 - minimal; 3 - mild secondary venous outflow and 4 - pronounced secondary venous outflow in one of the three pathways (VV, EV or DCV); 5– pronounced secondary venous outflow in two of the three pathways and 6 - maximum secondary venous outflow in all three pathways.

Volumetric flow rates through blood and CSF lumens were obtained using a semi-automated pulsatility based segmentation (PUBS) method for improved reliability [21]. The PUBS method utilizes velocity information throughout the entire time series to identify blood vessels or CSF lumen pixels, which in turn, results with a 4 folds increase in measurement reproducibility and increased measurement accuracy compared with manual delineation [21]. Time-dependent volumetric flow rate waveforms are obtained by integrating the flow velocities inside identified luminal cross-sectional area over all 32 phase contrast images representing one cardiac cycle. Flow waveforms were obtained for each of the four main cervical arteries (left and right internal carotid artery (LICA, RICA) and left and right vertebral artery (LVA, RVA)), for the primary venous pathways, the left and right internal jugular vein (LIJV, RIJV), and for the following secondary venous pathways: the vertebral veins (VV), epidural veins (EV), and deep cervical veins (DCV).

Total arterial blood flow, which is also the total cerebral blood flow (tCBF), is obtained by summation of the flow in the four arteries. Total jugular venous flow (tJVF) is defined as the sum of LIJV and RIJV. Secondary venous flow (SVF) is defined as the sum of the flow through the three secondary channels (VV+EV+DCV). Since total venous outflow is equal to tCBF, primary and secondary venous flow are also given as percentage of the tCBF to account for inter-subject variability. In addition, cervical CSF stroke volume, i.e., the volume of CSF that flows back and forth between the cranium and the spinal canal, was obtained by time integration of the CSF flow waveforms.

### Estimation of the Intracranial Compliance and Pressure

Details of the derivation of the intracranial compliance and pressure have been previously described [19], [22]. Briefly, based on the physical definition of compliance as a ratio of volume and pressure changes, intracranial compliance is estimated from the ratio of the maximal (systolic) intracranial volume (ICVC) and pressure fluctuations during the cardiac cycle (PTP-PG). The change in intracranial volume (ICVC) is obtained from the momentary differences between volumes of blood and CSF entering and leaving the cranium as shown in equations 1 and 2,
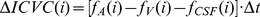
(1)

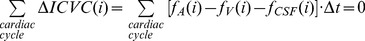
(2)where 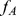
is arterial inflow, 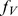 is venous outflow, and 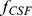 is the craniospinal CSF flow. Equation 2 states that in steady state, the intracranial volume is on average constant over an entire cardiac cycle. This condition is used to account for the unmeasured fraction of the total venous outflow.

The pressure change is derived from the amplitude of the CSF pressure gradient (PG) waveform obtained using the Navier-Stokes relationships between derivatives of velocities and the pressure gradient. An MRI equivalent of ICP (MRICP) is then obtained based on the reported inverse relationship between compliance and ICP [23]. Volumetric blood and CSF flow rate waveforms and derived parameters were obtained using a dedicated software tool (MRICP version 1.4.35 Alperin Noninvasive Diagnostics, Miami, FL).

### Volumetric Assessment of the Lateral Ventricles

The 3D T1 weighted images (MPRAGE) were used for assessment of the lateral ventricular volumes using 3D Slicer (v. 3.6.3, Surgical Planning Laboratory, BWH, Boston, MA) by placing two different regions of interests (ROI) in the left and right lateral ventricles. A used defined threshold was used to identify the ventricular boundaries. Where necessary, the ventricular ROIs were manually edited by a trained radiologist (A. P). The ventricle volume was then quantified by multiplying the number of voxels inside the ventricular region and the voxel size.

### Statistical Analysis

Linear mixed effects regression models with rank-transformed dependent variables were used to test for differences between the mTBI and the matched subjects’ clusters as rank transformation is beneficial for a small sample size. The intercept was allowed to vary by the matched subjects (random effects term). A two-step procedure proposed by Conover and Iman [24] was used in this work for the ranks conversion first followed by a parametric analysis (e.g., a linear mixed effects model) on the ranked data instead of on the original scales of the data. A non-parametric test (Mann-Whitney-U) was applied to test group differences of the visual scores for venous drainage patterns and for the ventricular volumes. Spearman’s rank correlation was used to test whether any of the hemodynamics and hydrodynamics parameters were correlated with time post injury. A p-value of <0.05 (two-sided) was considered statistically significant. Statistical analyses were performed with R (version 2.12.2) and SPSS (version 20.0), respectively.

## Results

### Venous Drainage

**Figure 1:**
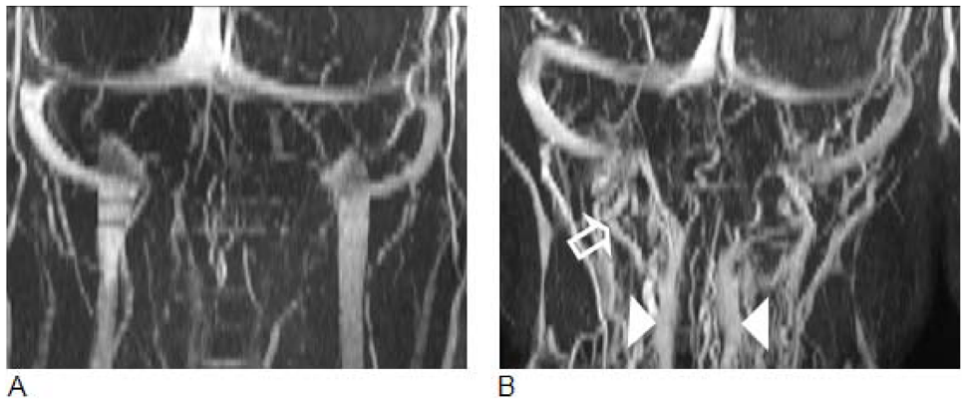
3D Maximum Intensity Projected MR venography images demonstrating cerebral venous drainage patterns in a control subject (A) and in a subject with mTBI (B). The internal jugular veins are well visualized in the control subject as the dominant outflow channel, while in the mTBI subject, the IJVs are not visualized and venous drainage occurs primarily through secondary veins, i.e., the epidural (filled arrows) and vertebral veins (empty arrow), which are well visualized.

Conventional MR sequences did not demonstrate any differences between the two cohorts and no visible abnormalities such as signs of bleeding, brain concussion or enlarged ventricles were seen. Volumes of the lateral ventricles were similar in the two groups (mTBI 18.6±6.3 ml vs. controls 18.2±10.5 ml; p = 0.351). In contrast, the MRV revealed significant differences between the two cohorts. Compared to control subjects, the mTBI cohort demonstrated reduced drainage through the IJVs associated with an increased venous outflow through secondary pathways which was demonstrated by a median grading of 4 for the mTBI group and 3 for the controls (p = 0.004). An example of cerebral venous drainage patterns in a control subject and in a subject with mTBI is shown in Fig. 1. The IJVs are well visualized in the control subject as the dominant outflow channel, while in the mTBI subject, the IJVs can hardly be distinguished from the dense network of draining secondary veins, i.e., the epidural and vertebral veins.

**Figure 2:**
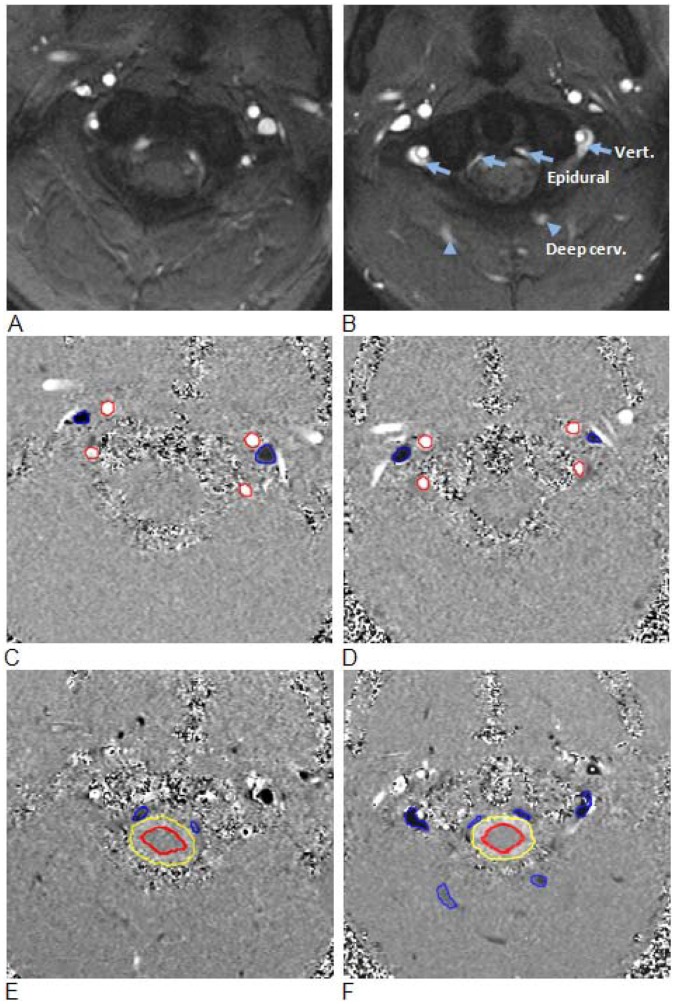
Examples of high and low velocity encoded phase contrast images from a control subject (left) and a subject with mTBI (right). A–B: Flow compensated magnitude images showing the bright signal from blood vessels. The augmented venous outflow through the epidural, vertebral veins (arrows) and the deep cervical veins (arrow heads) is well visualized. C–D: High-velocity encoding images used for measurements of arterial inflow and venous outflow through the jugular veins. E–F: Low-velocity encoding images used for measurements of the flow through the secondary channels (epidural, vertebral, and deep cervical veins) and the CSF flow. The lumen boundaries (red – arteries, blue- veins and yellow and red – CSF and cord) were identified using the PUBS automated segmentation method.

**Figure 3:**
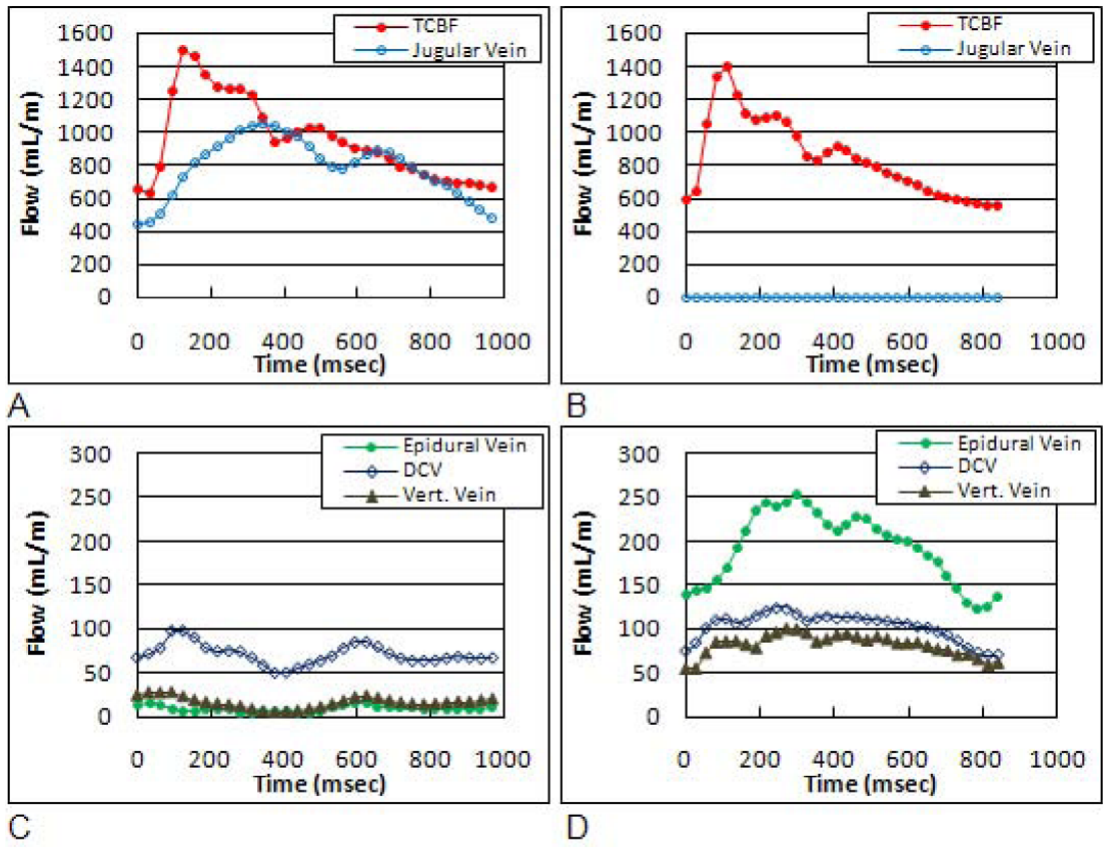
Derived volumetric flow waveforms obtained from a control subject (left) and a subject with mTBI (right). The total arterial inflow (TCBF) and total venous flow though the jugular veins are shown in the top (Fig. 3A and 3B), respectively. The measured venous flow through the epidural, deep cervical, and vertebral veins are shown in the bottom (Fig. 3C and 3D).

The quantitative evidence for reduced drainage through IJVs and increased secondary drainage is summarized in Table 1. Examples of magnitude and high and low velocity encoding phase images from a control and an mTBI subject are shown in Fig. 2. The blood and CSF lumen boundaries identified by the semi-automated pulsatility based segmentation method are overlaid on the phase images. Arterial flow toward the brain is shown in white, while venous outflow is shown in black. Derived tCBF and total jugular volumetric flow rate waveforms obtained from the segmented phase contrast images of a control subject and of a subject with mTBI are shown in Fig. 3a and Fig. 3b, respectively. Venous flow waveforms through the three secondary veins are shown in Fig. 3c, and Fig. 3d, respectively. Despite a high inter-individual variability in the amount of venous flow through the secondary veins, a significantly higher fraction of venous outflow occurs through these secondary veins in mTBI, compared to control subjects (12.3±11% vs. 5.5±3%; p<0.033). Consistently, the relative drainage through the jugular veins was significantly lower in mTBI (mTBI: 60.9±21% vs. controls: 76.8±10%; p = 0.01). The differences in venous drainage are not related to the magnitude of tCBF as there was no significant difference in the mean tCBF between the two groups (mTBI: 838±147 ml/min vs. controls: 779±112 ml/min; p = 0.439).

**Table 1:** Absolute and relative arterial inflow and venous outflow volumetric flow rates through the primary and secondary venous channels.

	Age	Gen.	TCBF	JVF	JVF	Secondary venous outflow (%)				MRV
ID	(Years)		(mL/m)	(mL/m)	(%)	*DCV*	*VV*	*EV*	Total	grading
MTBI_01	20	m	883	696	78.8	*0.4*	*0.0*	*1.9*	2.3	3
MTBI_02	22	f	966	564	58.4	*8.0*	*5.5*	*1.4*	14.9	5
MTBI_03	23	m	1020	718	70.4	*0.0*	*1.3*	*6.0*	7.3	3
MTBI_04	23	m	586	480	81.9	*0.0*	*0.0*	*5.8*	5.8	4
MTBI_05	23	f	797	484	60.7	*12.4*	*1.7*	*0.0*	14.0	4
MTBI_06	24	m	873	423	48.5	*1.5*	*22.4*	*2.5*	26.4	5
MTBI_07	25	m	694	355	51.2	*3.7*	*2.3*	*2.8*	8.9	4
MTBI_08	25	m	790	601	76.0	*1.3*	*0.2*	*0.0*	2.8	3
MTBI_09	27	m	1192	849	71.2	*1.7*	*3.3*	*0.9*	5.9	4
MTBI_10	27	m	704	381	54.2	*4.8*	*1.0*	*11.3*	17.1	5
MTBI_11	29	m	880	792	90.1	*0.0*	*0.0*	*0.0*	0.0	2
MTBI_12	29	m	791	355	44.9	*12.0*	*0.0*	*2.4*	14.4	5
MTBI_13	32	f	727	479	65.8	*10.0*	*0.0*	*0.7*	10.8	n.a.
MTBI_14	33	m	848	0	0.0	*10.9*	*9.6*	*22.7*	43.2	5
MTBI_15	49	f	826	511	61.9	*10.1*	*0.0*	*0.2*	10.3	4
CTR_01	18	m	911	599	65.7	*3.7*	*2.3*	*3.9*	9.9	4
CTR_02	22	f	575	382	66.4	*5.0*	*2.9*	*1.0*	8.9	4
CTR_03	23	m	688	490	71.2	*3.7*	*0.0*	*1.9*	5.6	n.a.
CTR_04	23	m	831	747	89.9	*1.8*	*0.0*	*2.5*	4.4	2
CTR_05	24	f	809	548	67.7	*1.3*	*0.0*	*0.0*	1.3	2
CTR_06	24	m	819	740	90.4	*0.0*	*0.0*	*3.1*	3.1	3
CTR_07	24	m	888	753	84.9	*0.0*	*1.4*	*3.0*	4.3	2
CTR_08	25	m	797	693	86.9	*0.0*	*0.0*	*2.5*	2.5	2
CTR_09	26	m	904	723	80.0	*7.4*	*0.0*	*0.0*	7.4	4
CTR_10	26	m	716	421	58.9	*6.0*	*0.0*	*5.8*	11.9	n.a.
CTR_11	27	m	842	706	83.8	*2.8*	*0.5*	*0.0*	3.3	2
CTR_13	30	m	591	444	75.2	*2.8*	*0.0*	*0.0*	2.8	3
CTR_12	32	f	687	453	66.0	*1.2*	*0.0*	*0.0*	1.2	2
CTR_14	32	m	917	774	84.3	*7.5*	*0.8*	*0.5*	8.8	4
CTR_15	48	f	718	583	81.2	*3.3*	*0.0*	*3.2*	6.5	3
Mean	mTBI		838	513	60.9	5.1	3.2	3.9	12.3	4.0
	CTR		779	604	76.8	3.1	0.5	1.8	5.5	2.8
p-value			0.44	0.22	0.01				0.03	0.004

JVF jugular venous flow, DCV = deep cervical veins, VV = vertebral veins, EV = epidural veins.

### Intracranial Compliance and Pressure

**Figure 4:**
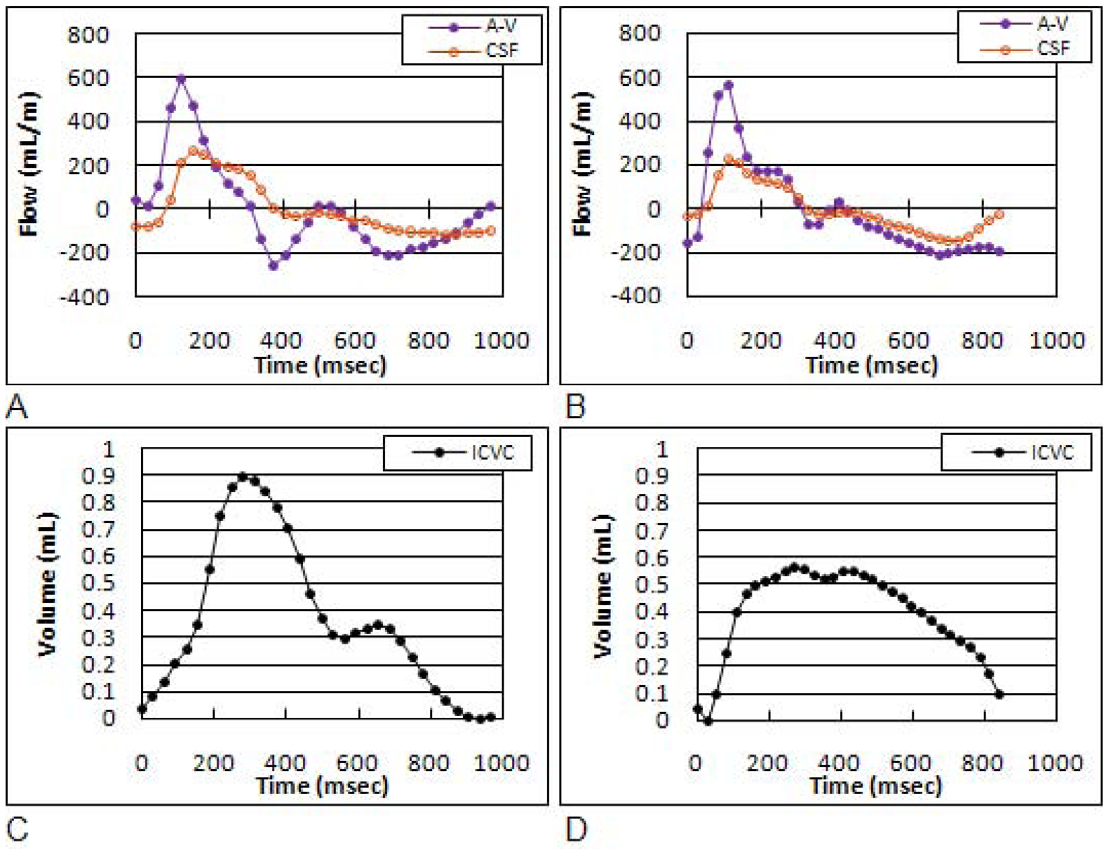
Derived volumetric flow rates and intracranial volume change waveforms obtained from a control subject (left) and a subject with mTBI (right). The arterial-minus-venous (A–V) and the CSF flow waveforms are shown in Fig. 4A and 4B. The Intracranial volume change during a cardiac cycle is shown in Fig. 4C and 4D, respectively. The CSF and the A–V waveforms are shown together to demonstrate the fact that the craniospinal CSF flow dynamics is driven by the net trans-cranial blood flow. The CSF waveform follows the A–V waveform more closely in the mTBI subject demonstrating the lower intracranial compliance compared to the matched control subject.

Examples of measured net trans-cranial blood flow (arterial inflow minus venous outflow or A–V) and cranio-spinal CSF waveforms from a control and an mTBI subjects are shown in Figures 4a and 4b, respectively. As can be seen, the CSF waveform “follows” the A–V waveforms more closely in the mTBI case, which is typical for low compliance. Since the CSF flow is driven by the A–V flow a tighter relationship indicates a less compliant intracranial compartment [25]. The waveforms of the intracranial volume change during the cardiac cycle of the control and the mTBI subjects are shown in Figures 4c and 4d, respectively. The Intermediate hydrodynamic parameters such as maximal or peak-to-peak pressure gradient (PTP-PG) and maximal intracranial volume change (ICVC) and MR estimate of intracranial pressure (MRICP) values are summarized in Table 2. While no statistically significant difference between the two groups was found for the PTP-PG (mTBI 0.04±0.01 mmHg/cm vs. controls 0.04±0.01 mmHg/cm; p = 0.66) and the ICVC (mTBI 0.48±0.1 ml vs. controls 0.61±0.2 ml; p = 0.07), a trend toward a lower maximal volume change was seen in the mTBI group. In contrast, the intracranial compliance index was significantly lower (mTBI 5.8±1.4 vs. controls 8.4±1.9; p<0.0007), and consequently MRICP was significantly higher (mTBI 12.5±2.9 mmHg vs. controls 8.8±2.0 mmHg; p<0.0007) in mTBI. Statistical significance was not derived by outliers. The higher MRICP values in the mTBI cohort were all within the normative range.

**Table 2:** MRI derived hydrodynamic parameters.

	Age	Gen.	CSF SV	PTP-PG	ICVC	MRICP
ID	(years)		(ml)	(mmHg/cm)	(ml)	(mmHg)
MTBI_01	20	m	0.70	0.053	0.58	14.9
MTBI_02	22	f	0.51	0.054	0.41	16.4
MTBI_03	23	m	0.72	0.043	0.49	10.6
MTBI_04	23	m	0.47	0.042	0.36	11.7
MTBI_05	23	f	0.70	0.034	0.54	9.1
MTBI_06	24	m	0.65	0.048	0.53	10.6
MTBI_07	25	m	0.30	0.030	0.53	9.6
MTBI_08	25	m	0.98	0.036	0.69	8.7
MTBI_09	27	m	0.66	0.053	0.64	12.1
MTBI_10	27	m	0.21	0.053	0.31	16.7
MTBI_11	29	m	0.54	0.047	0.49	14.9
MTBI_12	29	m	0.56	0.050	0.44	15.5
MTBI_13	32	f	0.60	0.030	0.33	11.2
MTBI_14	33	m	0.59	0.045	0.55	9.2
MTBI_15	49	f	0.54	0.034	0.26	15.7
CTR_01	18	m	0.84	0.054	0.82	10.4
CTR_02	22	f	0.41	0.031	0.50	9.3
CTR_03	23	m	0.60	0.049	0.57	9.1
CTR_04	23	m	0.50	0.031	0.77	5.8
CTR_05	24	f	0.40	0.022	0.27	10.7
CTR_06	24	m	0.69	0.069	0.74	10.4
CTR_07	24	m	0.63	0.023	0.56	7.4
CTR_08	25	m	0.41	0.054	0.52	13.8
CTR_09	26	m	0.56	0.056	0.97	7.5
CTR_10	26	m	0.53	0.041	0.64	9.2
CTR_11	27	m	0.60	0.058	0.85	7.6
CTR_13	30	m	0.41	0.030	0.44	6.7
CTR_12	32	f	0.48	0.031	0.44	8.0
CTR_14	32	m	0.80	0.042	0.83	6.7
CTR_15	48	f	0.21	0.023	0.27	8.6
Mean	mTBI		0.58	0.043	0.48	12.46
	CTR		0.54	0.041	0.61	8.77
p-value				0.66	0.07	0.0007

SV = stroke volume, PG = pressure gradient, ICVC = intracranial volume change, MRICP = MR derived intracranial pressure.

Finally, Spearman’s rank correlation did not reveal association of the hemodynamics and hydrodynamics parameters with time since injury. Lowest P value of 0.13 was obtained for the relative venous drainage through the smaller secondary channels.

## Discussion

This study employs MR imaging methods to explore whether mTBI is associated with altered hemodynamics and hydrodynamics. Qualitative and quantitative assessments by MR venography and MR velocity encoded arterial inflow and venous outflow measurements demonstrate significant differences in venous drainage pattern between the asymptomatic mTBI subjects and the control cohort. mTBI is associated with a significantly smaller fraction of the cerebral blood drainage through the IJVs, i.e., the primary drainage pathway in the supine posture. On average, only 60.8% of the cerebral blood flow leave the brain though the IJVs in the mTBI subjects, compared to 76.8% in the matched subjects who did not report mTBI. The IJV drainage fraction in the control subjects measured in this study is in excellent agreement with a previous study of healthy subjects that reported approximately 75% of the cerebral blood drain through the IJVs in supine posture and 40% in the upright posture [14]. As expected, the smaller fraction of venous drainage through the IJVs in mTBI was associated with increased venous drainage through secondary venous pathways, which drain the neurocranium in parallel to the IJVs [15], [18]. Increased drainage through secondary veins usually occurs in the upright posture, partly due to complete or partial collapse of the IJVs. The reason for increased venous drainage through the secondary veins in supine in subjects after mTBI is unclear at this time. Yet, similar changes in supine have previously been reported in patients with idiopathic intracranial hypertension [26] and chronic migraine [27].

The physiology of the cerebral venous system and its relationship to the intracranial hydrodynamics is still not fully understood. The concept of venous hemodynamics is complicated by the fact that veins are collapsible blood vessels, which are characterized by marked changes in their cross sectional configuration in response to even a slight change in transmural pressure [18]. Increased secondary venous drainage in the supine position has also been reported in idiopathic intracranial hypertension [26], thus potentially linking increased secondary venous drainage in supine, which represents high impedance for venous outflow, with reduced compliance and higher ICP. An association between higher ICP and changes in venous flow characteristics has previously been documented in mechanical models of cerebral drainage and in patients [28].

A significantly lower intracranial compliance and higher MR-derived estimate of ICP (MRICP) were found in the mTBI cohort. The mean value of 8.8 mmHg found in the control group is in excellent agreement with a mean value of 9.6 mmHg previously reported in a study of 23 healthy young adults [29]. On average, the MR-estimate of ICP in mTBI was 12.5 mmHg, which is approximately 3.7 mmHg higher than the mean MRICP value found in the control group. Interestingly, neither the maximal volume change nor the pressure gradient changes were statistically different between the two cohorts. Yet, the ratio of these parameters, i.e., the compliance index, did reach a strong statistically significant difference (p = 0.0007) between the two cohorts. Statistically, the variability of the ratio of two other parameters is larger than the individual variability. The fact that the intracranial compliance index, did reach statistical significance supports the reliability of the findings of reduced intracranial compliance in mTBI. In fact, effects size for the compliance index and the MRICP variables of 1.5 and 1.7, respectively are among the largest reported for group difference between old mTBI injured patients and controls.

Another important observation is the persistence of the hemodynamic and hydrodynamic changes following the mTBI. A study employing MR diffusion techniques following moderate traumatic brain injury demonstrated persistent changes in water diffusivity even at 6 months following trauma [30]. A more recent study by MacDonald et al. reports abnormal findings at a much higher rate than is expected by chance in scans performed 6 to 12 months following enrolments in military personal with clinical diagnosis of mild, uncomplicated traumatic brain injury [31]. It is therefore plausible that diffuse parenchymal changes that do not fully resolve over time, contribute to the reduced intracranial compliance through changes in brain volume.

Although potential mechanisms behind the altered hemodynamics and hydrodynamics found in mTBI cannot be established based on the data presented in this study, several factors may potentially contribute based on previously published data on animal models and humans. Potential mechanisms underlying the observed altered venous drainage and reduced intracranial compliance in mTBI include:

### 

#### a) Structural changes in the brain microvasculature following mild traumatic brain injury

It is known from animal studies that long lasting changes occur in the endothelium and in the perivascular astrocytes of baboons after lateral head acceleration [12]. Moreover, a recently published study in 12 professional boxers found an impaired dynamic cerebral autoregulation and a reduction in the cerebrovascular reactivity to changes in carbon dioxide [32]. These changes could potentially be linked to the reduced overall intracranial compliance through reduced vasculature compliance and to the increase in secondary venous drainage.

#### b) Post-inflammatory changes following mild traumatic brain injury

Increases in inflammatory cytokines (e.g. IL 2, IL 6 and TNF alpha) have been reported following mTBI. A recent study by Jin et al. documented changes in inflammatory cell marker expression and cellular infiltration in a controlled cortical impact model in mice [33]. Migration of inflammatory cells is known to mostly occur in the venous vasculature [34]–[36]. Recent experimental studies have observed increased leukocyte-endothelium interactions in venules after TBI [34]. The resulting local inflammatory response may subsequently induce the formation of microthrombi occluding cerebral venules. Inflammatory responses might influence the endothelial cells and the local production of vasoactive substances such as NO and endothelin, which in turn can influence cerebral hemodynamics. An inflammatory reaction with a potentially increased in vessel walls rigidity may lead to or contribute to the observed altered venous hemodynamics and a lowered intracranial compliance. Yet, it is important to note that altered venous drainage is not specific to mTBI. Recent reports documented altered venous drainage, without a change in intracranial compliance, in migraine headaches and multiple sclerosis [27], [37].

The main limitation of the study is related to the self-reporting used for including the subjects with mTBI. Therefore, there is a lack of objective verification of the nature of the mTBI. However, the inclusion criteria were based on the guidelines and conceptual definition developed by the Centers for Disease Control and Prevention [1]. Another limitation relates to the studýs cross-sectional design and the large heterogeneity of time span since the trauma. This study design does not provide information regarding the timing and the rate at which these changes occur following the trauma. However, the persistence of the changes found in this study is consistent with existing literature demonstrating that mild TBI leads to irreversible changes. These preliminary results warranted further investigations including larger cohorts and specifically designed to address the time evolution and magnitude of these alterations in relations to the elapsed time after trauma and trauma severity, respectively.

In conclusion, the current study provides imaging-based evidence of a chronically reduced cerebral venous drainage through the internal jugular veins, an increased drainage in secondary venous channels with a concomitantly mild decreased intracranial compliance and mild increased intracranial pressure in supine subjects with a history of mTBI. As all mTBI subjects were free of symptoms at the time of the scan, the observed changes seem to have no apparent impact on brain function. The precise mechanism of these alterations remains to be elucidated. However, these results suggest that MR measures of compliance or ICP are potentially a sensitive marker for detecting hemodynamic and hydrodynamic changes associated with mTBI.
