# Correction: MRI Evidence for Altered Venous Drainage and Intracranial Compliance in Mild Traumatic Brain Injury

**DOI:** 10.1371/annotation/bb7d0e00-ae4b-4ddc-af11-020d75a45a8e

**Published:** 2013-12-30

**Authors:** Andreas Pomschar, Inga Koerte, Sang Lee, Ruediger P. Laubender, Andreas Straube, Florian Heinen, Birgit Ertl-Wagner, Noam Alperin

The first two authors, Andreas Pomschar and Inga Koerte, should be noted as contributing equally to this work. 

